# Cultural hierarchies in health: Does inherited sociocultural position (biraderi) shape diet and nutrition among British Pakistani children? Protocol for a mixed-methods study

**DOI:** 10.1371/journal.pone.0305556

**Published:** 2024-06-17

**Authors:** Komal Bhatia, Hannah Intezar, Parveen Akhtar

**Affiliations:** 1 Institute for Global Health, University College London, London, United Kingdom; 2 School of Social Sciences, University of Bradford, Bradford, United Kingdom; 3 Politics, History and International Relations, Aston University, Birmingham, United Kingdom; Public Library of Science, UNITED STATES

## Abstract

This study aims to explore links between *biraderi*–a form of identity-based social grouping and stratification which cuts across religions among South Asians–and infant and child nutrition among British Pakistanis using data from the Born in Bradford cohort study. The study will entail a mixed-methods approach to (i) develop an operational framework of biraderi for epidemiologic analyses and apply it to longitudinal data from the Born in Bradford cohort study, (ii) quantify and describe child nutrition and dietary patterns for biraderi sub-groups, and (iii) investigate whether known mechanisms of identity-based segregation, graded inequality, and network effects operate through diet and nutrition in the UK. Using Krieger’s ecosocial theory as an integrative framework we will (iv) re-conceptualise and interpret the role of biraderi / caste in the social construction and embodied experience of how infants and children eat in the UK. Following a literature review on biraderi and health, we will convene and consult a lay consultation group in Bradford through focus groups and academic experts through a Delphi study to guide planning, implementation, interpretation and dissemination of our secondary data analysis. In addition to being the first study to look at biraderi-based nutritional inequalities in the UK, our study is innovative in that we will formally involve experts and users in the design and interpretation of our quantitative analyses. Findings will be applicable in any part of the world where children experience disadvantage linked to sociocultural hierarchy and identity. Our findings will be of particular use in (i) identifying women and children at particular risk of suboptimal breastfeeding practices, poor complementary feeding, and unhealthy diets in primary school in the UK, and (ii) elucidating the sociocultural pathways through which inequalities in population health nutrition outcomes are expressed.

## Introduction

This study aims to explore links between *biraderi*–a form of identity-based social grouping and stratification which cuts across religions among South Asians–and infant and child nutrition among British Pakistanis using data from the Born in Bradford cohort study. Ethnic minorities’ higher risk of ill health compared to the White British population has been explained by social structural variables (poverty, deprivation) and occupational hierarchy [1]. Little research has looked at the role of inherited and embodied socio-cultural hierarchies within minority communities that are themselves related to health.

Biraderi has links with the construct of caste and encompasses a broader group of concepts related to historical classes in Hinduism (*varna*) and smaller, hierarchically ranked regional birth groups (*jati*) across religions [2]. Biraderi, *zat* or *zaat* is commonly used by British Pakistanis to express kinship grouping, occupation, and place of origin, but also finds negative expression as a form of social ranking [3, 4]. Functionally, biraderi networks operate in the social, economic, and political spheres. Migration chains from South Asia to the UK can be traced along biraderi lines, which subsequently served as informal welfare networks amongst the diaspora, helping to find work and accommodation, as well as offering psychological and financial support [4–6]. More recently, biraderi continues to shape the political experiences of British South Asian Muslims [7, 8]. Exploratory research points to biraderi’s possible impacts on caring responsibilities among British Pakistanis [9], but a social epidemiologic study in the UK is long overdue.

Empirically, in South Asia and among the UK diaspora, caste plays a role in shaping access to services and opportunity, experiences of discrimination and prejudice, and the process of socialising children into caste identity [2]. Food has historically been a tool to perpetuate hierarchy through prescriptive diets and food taboos, while 20^th^ Century processes of affirmative action and globalisation in South Asia have made the relationship between food and caste more complex [10]. Adequate food and nutrition in infancy and childhood are critical to health across the life course [11]. In India, caste-based discrimination in access to child nutrition programmes [12] and graded inequalities in food consumption [13] persist, but analogous research among South Asians in the UK does not yet exist. There is then, a clear gap in knowledge around the impacts (if any) of caste on child nutrition in the UK. This leads to the central question of this research: Does biraderi shape child nutrition in the UK as caste does in India?

The Born in Bradford (BiB) cohort study [14] is one of the few epidemiologic resources worldwide with granular data on biraderi for c. 4000 families of South Asian origin. Biraderi has only featured in one genetic analysis [15] while longitudinal infant and young child nutrition data have yielded several insights [16, 17], indicating BiB’s potential to unmask identity-based nutritional inequalities among children in the UK.

### Aims and objectives

Our aim is to examine how biraderi is related to British Pakistani women’s breastfeeding practices and their children’s diets, and to understand its implications for health inequalities as a wider public health issue from an ecosocial perspective.

We will achieve this through four objectives:

1. Develop an operational framework of biraderi for epidemiologic analyses and apply it to longitudinal data from the Born in Bradford cohort study to:
2. Quantify and describe child nutrition and dietary patterns for biraderi sub-groups, and
3. Investigate whether known mechanisms of identity-based segregation, graded inequality, and network effects operate through diet and nutrition in the UK.
4. Reconceptualise and interpret the role of biraderi / caste in the social construction and embodied experience of how infants and children eat in the UK

## Methodology

Our approach is shaped by the challenge of employing biraderi as an ordered categorical variable with which to investigate health and nutritional inequalities either along a gradient or compared to a ‘highest’ or ‘reference’ group, as is possible for income or education in the UK or for caste in India. A clear definition of social groups and ranking is important for operationalising analyses of inequalities when using an ordered categorical variable [18]. There is no standard academic or legal definition and ordering of biraderi groups, and academic texts are unlikely to map exactly onto lay perceptions or lived experiences that may have changed over time and across generations. We will therefore create an operational framework for our secondary data analysis of BiB data.

Our methodology involves combining consensus building to develop a framework on biraderi with secondary data analysis of the BiB cohort. We will work closely with (i) the BiB study’s statisticians, data managers, and nutrition lead to obtain and code datasets; and convene (ii) an Expert Consultation Group, and (iii) a Lay Consultation Group to guide planning, implementation, interpretation and dissemination of our secondary data analysis.

We will conduct a mixed-methods study with four inter-linked components:

1. Literature Review on biraderi and health
2. Delphi study with an Expert Consultation Group
3. Focus Groups with a Lay Consultation Group
4. Secondary analysis of Born in Bradford cohort datasets

**Fig 1:**
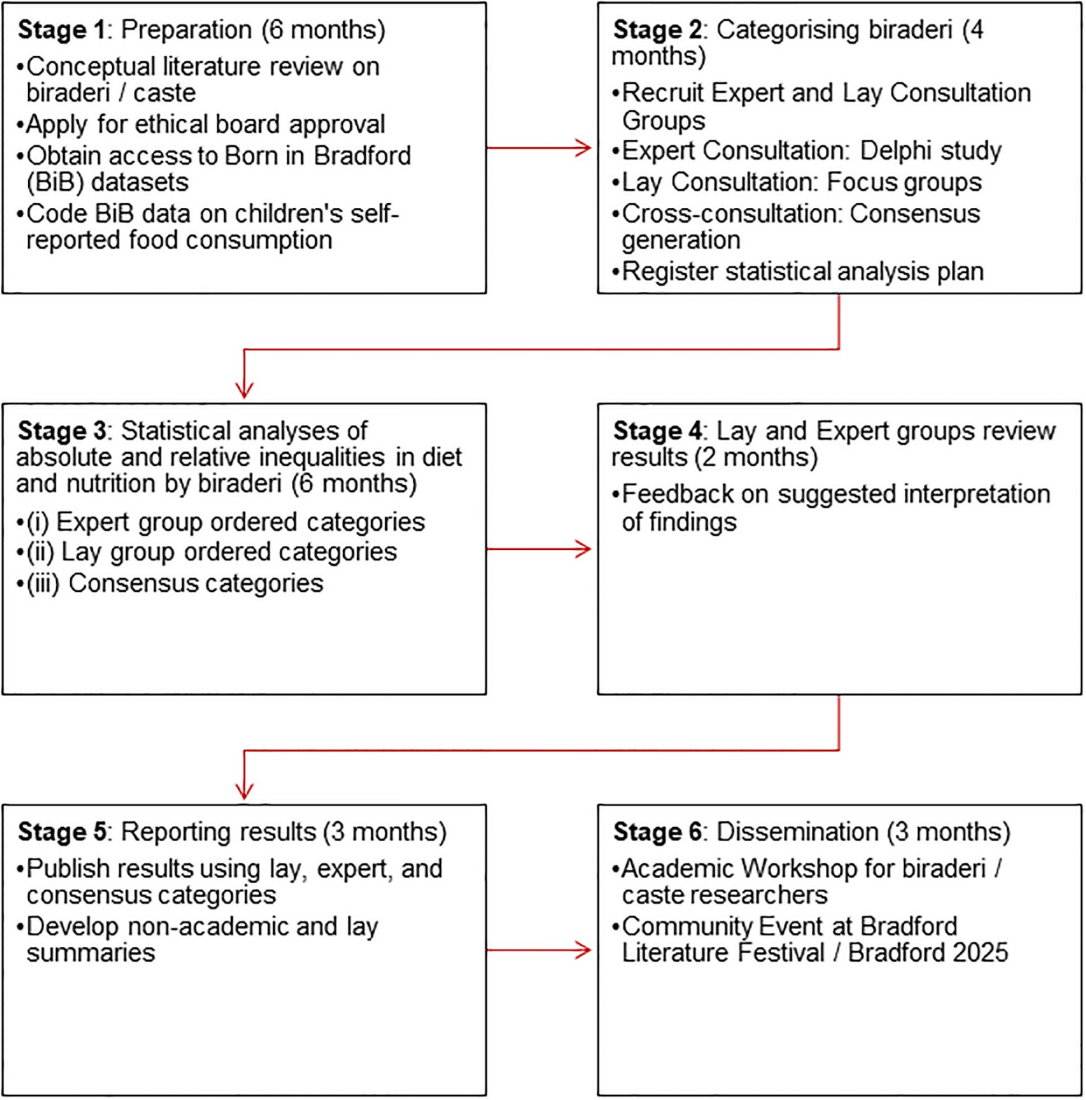
Proposed methodology.

We will conduct an online Delphi study [19] with academic experts and focus groups with lay members of the Bradford community to generate consensus on the definitions of biraderi groups and ordering. For the Delphi study, we will recruit an Expert Consultation group by identifying individuals in the bibliography of our conceptual review. We will conduct and report the Delphi study using the CREDES checklist [20]. For our participatory research with lay members, we will conduct focus groups with 20 male and 20 female Bradford residents of South Asian origin who identify as belonging to a biraderi over 3–4 in-person sessions. We will approach students from the University of Bradford to recruit members for our Lay Consultation Group from across the biraderi groups as well as local mosques. After each consultation group has developed a ranking of biraderi groups based on their respective processes, we will hold a cross-consultation to allow each group to comment on the other’s conclusions and to use this to generate a consensus ranking. At the end of this exercise, we hope to have (i) expert, (ii) lay and (iii) consensus rankings of biraderi to use in three separate sets of analyses. After we have completed secondary analysis of Born in Bradford data, we will give the Lay and Expert Consultation Groups a chance to comment on our findings and seek their advice on interpretation, messaging and dissemination of findings. We will achieve this in six stages over 24 months. (Fig 1).

Due to the iterative and cumulative nature of our specific analysis strategies for the BiB dataset, we will record specific outcomes and decisions from our Lay and Expert Consultation Groups as well as a-priori and post-hoc statistical analysis plans on Open Science Framework (OSF). Our OSF page will be updated regularly during the project to bring together linked components, and can be viewed here: https://osf.io/bp4hz/?view_only=b0b2495b716b43c0a29f30dc04c119c9.

Our study has received ethical approval from the University College London Research Ethics Committee (Project ID: 7403/004) and the University of Bradford Research Ethics Committee (Project ID: E1193).

## Theoretical frameworks

Our study is guided by theoretical ideas in social epidemiology and development studies. We will draw on (i) statistical methods in social epidemiology [21], (ii) the wider literature on mechanisms through which caste / biraderi operates [22], and (iii) Nancy Krieger’s ecosocial theory [23–25].

### Statistical methods in social epidemiology to measure health disparities

As our starting point, we refer to Harper and Lynch’s guidance on operationalising analyses of health inequalities in social epidemiology [21]. They suggest that a priori decisions about analytic processes are important in order to make substantive judgements about health inequalities [18, 26]. These decisions include selecting measures and metrics related to absolute vs relative measures of inequality, population weighting, use of reference points or groups for comparison, and including all groups or only best-and-worst extremes.

Measure of health disparity can be of four types, (i) total disparity, (ii) social-group disparities, (iii) average disproportionality and (iv) measures that combine health disparity and average health. Some, but not all of these measures, can be used for groups where ranking or ordering exists.

In their proposed methodological approach, group specific outcomes should be examined in tables or graphs to identify patterns and heterogeneity, and the situation of specific sub-groups. For pair-wise comparisons, suitable absolute and relative indicators of disparity should be selected, but absolute measures should be prioritised. For comparison across all groups, summary measures should be selected based on whether social groups are ordered or un-ordered. Measures for ordered social groups should be sensitive to gradients in health outcomes, flexible to incorporating differing levels of inequality, and account for changes in underlying distributions over time. Measures for unordered social groups should allow decomposition into between-group and within-group components for continuous outcomes.

While Harper and Lynch’s epidemiological approach provides sufficient technical guidance on how to work with data on social groups and operationalise hypothesised relationships between variables, specific guidance on whether and how to order or rank groups is perhaps beyond its scope.

### Caste and development

The contemporary role of caste as a persistent structure of discrimination and disadvantage is explained at length in a 2018 review focused on the Indian context by David Mosse [22], which allows us to translate key characteristics of caste processes into mechanisms linking caste to health that can be investigated epidemiologically.

Mosse argues that caste operates as a shadow indicator in international development, excluded from larger frameworks such as the Sustainable Development Goals, but tracked by advocacy groups that aim for its inclusion as a form of discrimination. Its position as an issue rooted in religion and historical disadvantage means it does not have a natural position in national economic or health planning policy, and remains an internal matter of little relevance to global development.

Salient characteristics of caste that make it important for global policy are forms of occupational ranking, exclusion and enclosure, network effects, graded inequality, and stigmatisation. Caste involves processes of both categorical exclusion and opportunity hoarding, and it persists because of its advantages at the expense of discrimination experienced by others.

Empirical evidence points to the need to have informed discussions of caste inequalities, but methodological work on testing these statistically remains scarce.

While there is a considerable development studies literature on caste as a structure of segregation, discrimination, and disadvantage in South Asia [22, 27, 28], social epidemiology has only recently, and tentatively, addressed caste [29]. The development and social epidemiologic frameworks will provide an operational starting point to look at diet and nutrition as an expression of biraderi / caste, but these perspectives still lack the necessary theoretical ideas to help us (re)-conceptualise and interpret the role of biraderi / caste in the social construction and embodied experience of how infants and children eat.

### Krieger’s ecosocial theory

Nancy Krieger’s ecosocial theory [23–25] offers an integrative epidemiologic basis to conceptualise the links between biraderi and diet through its core ideas of

(i) embodiment
(ii) pathways of embodiment
(iii) the dynamic and cumulative interplay between exposure, susceptibility, and resistance
(iv) accountability and agency

Ecosocial theory has been applied widely, to explore breastfeeding [30] and food insecurity [31] among minorities in North America, discrimination in Israel [32] and wellbeing in Ghana [33]. We are interested in using ecosocial theory as a framework to extend biraderi to social and nutritional epidemiology.

We intend to apply Krieger’s Ecosocial Theory and its four core ideas across the study *as a whole* in the following three ways: (1) Statistical analysis will enable us to understand whether the three pathways linking biraderi to child nutrition can be viewed as pathways to embodied inequalities should these exist (Krieger’s ideas i and ii); (2) Lay and expert consultation groups’ contribution to data analysis and interpretation will help us elucidate how biraderi grouping shapes the interplay between exposure, susceptibility, and resistance to poor child nutrition and diet (Krieger’s idea iii); (3) Community events and activities will stimulate dialogue around accountability and agency (Krieger’s idea iv). Since Krieger’s theory is intended to be used as an *integrative* theory, it will help us bring together all the components of the study into one clear framework embedded in social epidemiology.

## 1. Literature review on biraderi and health

We will pre-register and regularly update our detailed review protocol on OSF to enhance the reproducibility of our review methods, but an overview is below.

### Review aims and research questions

Our project will be supported by a literature review on biraderi and its links with health. The two key aims of our review are to (i) summarise prior knowledge on biraderi as a social indicator and (ii) aggregate or integrate data on its relationship with health.

To summarise prior knowledge on biraderi categorisation schema and domains of influence, our review needs to be descriptive in nature, broad in scope, rely on a representative set of empirical sources, and requires little or no quality appraisal. Summarising literature will allow us to create an operational framework for biraderi as a viable epidemiologic exposure of interest.

To aggregate data on the importance of biraderi for multiple life domains and the complexity of the mechanisms through which it operates, it needs to be broad in scope but rely on a comprehensive set of empirical and conceptual sources, with little or no quality appraisal, and to conduct content or interpretive analysis. Aggregation and integration will allow us to expand the conceptual basis for biraderi as a critical determinant of health.

We will address four research questions:

1. What does the concept of biraderi mean from a sociological perspective?
2. How are biraderi groups and sub-groups described and classified?
3. In which health domains is biraderi known to have influence?
4. What pathways and mechanisms of influence between biraderi and health have been proposed in the literature and how does this compare with the literature on caste?

### Identifying relevant literature

The review will be multidisciplinary, reflecting the make-up of the study team and recognising that the topic of biraderi cuts across disciplines.

We will include theoretical and empirical material on biraderi, recognising that theoretical articles or book chapters may include empirical examples and empirical studies may explain theory at length. We will use diverse source material, drawing on the personal collection of literature on biraderi or caste maintained by the study team alongside electronic searches and citation tracking.

Study participants will be individuals or communities who identify or belong to a biraderi group, with a comparison group drawn from within biraderi sub-groups or from external populations. We are interested in studies set in South Asia or diaspora communities, but we are particularly interested in studies set in Pakistan as the source population for many BiB participants of British-South Asian origin. Relevant studies should focus on biraderi as a social indicator. For empirical studies, the role of biraderi should be examined in at least one domain (for example, politics, access to healthcare, life expectancy, educational or employment opportunities).

We will identify relevant study in two stages.

First, we will identify a list of seminal texts on biraderi from a preliminary electronic search as well as our personal libraries, based on which we will develop a framework for a more in-depth search. Preliminary terms to guide the search will include biraderi, biradari, biraaderi, kin, kinship networks, Pakistani diaspora, ethnic kin, ethnic ties, and diaspora kinship, familial ties.

Second, we will use a systematic search strategy to identify a larger set of empirical and theoretical studies on biraderi.

### Selecting studies

Inclusion and exclusion criteria will not be rigid, and we will adopt a flexible approach to meet the aims of the review. Criteria will be developed and revised based on new and ongoing familiarity with the topic through reading seminal texts as well as a subset of empirical studies. For feasibility, we will not conduct independent or duplicate screening of search results or data extraction.

### Data extraction

For theoretical and conceptual material identified through our list of seminal papers, we will proceed to summarising key concepts and ideas from individual texts, and deem data extraction unnecessary.

For material identified through the systematic search, we will adopt a pragmatic approach and limit data extraction to key pieces of information from studies that will support our consultation exercises, focussing on study setting, population characteristics, biraderi categories or schema, and domains in which biraderi has a role.

### Collating, summarising and reporting results

We will summarise and synthesise seminal texts through a subjective process of induction and interpretive collating. For theoretical and empirical literature identified systematically, we will conduct content and simple frequency analysis of key biraderi categorisation scheme and domains of influence, focussing on health. We will not conduct quality appraisal, but will critically evaluate and discuss the literature at the write-up stage and cross-reference interpretations across the team.

The review will provide three practical outputs which will feed into work with the Expert and Lay Consultation Groups and secondary analysis of BiB data.

First, we will produce a list of experts on biraderi through bibliometric analysis of literature included in our review to use as a sampling frame for the Delphi study and to identify and contact eligible participants. We will also use our collective academic knowledge of the research area to identify key experts.

Second, we will summarise background information on the history, meaning and role of biraderi as a social indicator in South Asia and the UK, and use this to inform discussions with the two Consultation Groups as well as planning statistical analyses and interpreting findings.

Third, we will use the list of biraderi categorisations, ranking or ordering in the existing literature as a starting point for the ranking component of the Delphi study and focus groups, by presenting these ideas to the participants and offering them the opportunity to build on these in the consultation.

## 2. Expert consultation group—Delphi study

### Aims and objectives of the expert consultation group

The overall aim of the Expert Consultation Group exercise is to involve academic experts on the topic of biraderi in the UK and internationally in our study. The Group will guide the study design and interpretation of results of our research on biraderi and its relationship with infant and child nutrition.

Through involvement in the study design, the Expert Consultation Group will create an operational framework of biraderi to use in statistical analysis, by using the 15 biraderi groups encoded in the Born in Bradford cohort to develop categories and / or ranking schema. This will be achieved through a Delphi study, which will also involve providing feedback on results of a parallel group run through focus groups discussions with a Lay Consultation Group, to potentially create a consensus ranking / grouping for biraderi.

Involvement in the interpretation of results will include a chance to provide feedback on results produced using the operational framework created by the Lay and Expert Consultation Groups, and to discuss the meaning and relevance of our findings for the study of biraderi and for health among South Asian and diaspora communities.

We will achieve this through a Delphi study with a minimum of four rounds, with the following objectives:

Round 1: Gather information on expert panellist’s understanding of the concept of biraderi and collect data on individual ranking of the 15 biraderi sub-groups in the BiB study
Round 2: Seek panellists’ comments on the analysis of ranking data generated in Round 1 and comment on the ranking developed by the Lay Consultation Group
Round 3: Seek individual panellists’ interpretation of the study team’s analysis of children’s food practices by biraderi groups
Round 4: Seek feedback on other panellists’ interpretation of the study findings as well as that of the Lay Consultation Group

### Participants

Eligible participants will be academic experts who have co-authored or contributed to at least one publication that addresses the topic of biraderi, encompassing books, monographs, journal articles, policy briefs, and research reports. Experts can be located anywhere in the world, and we are particularly interested in experts from Pakistan and India. By including participants based outside the UK, specifically in South Asian countries where researchers on biraderi have an active academic community, we would enable cross-national dialogue and attempt to ‘de-colonise’ this topic. Allowing researchers in South Asia to shape the design and interpretation of research in the UK, on a UK sub-population, would invert the power dynamic normally observed in global health and epidemiology.

We anticipate having a list of c. 50 potential participants to recruit from, of whom about 10 may decline. Therefore, we are aiming for 40 participants, and expect that 30 will contribute to the final consensus generation exercise. There will be no limit on the number of participants from a particular setting, though we will aim to have a gender-balanced panel. The same set of participants will be invited to the four Delphi rounds, and so we are aiming for a 50% retention rate in Rounds 3 and 4 to ensure content validity.

### Recruitment

We will draw up a list of experts on biraderi during our literature review. The list will serve as our sampling frame and include those identified through bibliographies and author lists of relevant literature, as well as experts who are already known to the study team through background reading and prior knowledge. Potential participants will be approached via email by the PI. We anticipate being able to obtain experts’ email addresses through their publicly available organisational profile or contact details on publications. We will email each potential participant directly and explain the purpose of our project and the Delphi study. We will explain what participation would entail, provide a timeline for each Delphi round, and share a Participant Information Sheet as an attachment or in the body of the email itself. Questions about the study will be answered by email. For those who indicate that they would like to participate, we will then share an individual personalised link to an online consent form. The recruitment period will run from 1 June 2024 to 1 June 2025.

### Consent

The Delphi process relies on anonymity within the expert group, such that researchers know who participants are, but participants do not know who else is participating in the study. This reduces the risks normally associated with group-based data collection methods such as focus groups, where it is impossible for researchers to ensure absolute confidentiality if personal information provided to the group is disclosed by participants to those outside the group.

Once participants have agreed to participate via email, we will send them a secure link to a MS Forms page on which they can read the consent form and ‘sign’ their consent by confirming a tick-box and entering their name. After this, they can proceed to the online form to participate in Round 1 of the Delphi study.

Due to the cumulative nature of data collection and iterative analysis feeding into successive Delphi rounds, participants will be able to withdraw from the study at any time, but they will not be able to withdraw data already collected in a previous round. Participants can withdraw from subsequent rounds up to two weeks after each round.

### Methods

We will conduct the Delphi study online using internet-based surveys in Microsoft Forms.

Rounds 1 and 2 will take place four weeks apart in the study design phase. Round 1 will entail individual data collection on experts’ ideas and views on biraderi as well as feedback on categorising and ranking biraderi sub-groups. Individual data from Round 1 will be collated and analysed by the study team to produce summary information and rankings that panellists can comment on in Round 2 to arrive at a consensus ranking.

After this, there will be a break of up to 6 months while the study investigators implement the categorisation and ranking schema in the planned analysis of Born in Bradford Data. In Rounds 3 and 4, panellists will give feedback on the study results, first individually without any information on other panellists’ responses (Round 3), and then will have a chance to comment on other panellists’ interpretation and the views of the Lay Consultation Groups (Round 4).

## 3. Lay consultation group—Focus groups

### Aims and objectives of the lay consultation group

The overall aim of the Lay Consultation Group exercise is to involve members of the 1^st^ to 4^th^ generations of British Pakistanis in Bradford in the study design and interpretation of results of our research on biraderi and its relationship with infant and child nutrition.

Through involvement in the study design, the Lay Consultation Group will create an operational framework of biraderi to use in statistical analysis, by using the 15 biraderi groups encoded in the Born in Bradford cohort to develop categories and / or ranking schema. This will be achieved through focus groups discussions with Lay Consultation Group members, which will extend to also providing feedback on results of a parallel exercise run as a Delphi study with an Expert Consultation Group of academics to potentially create a consensus ranking / grouping for biraderi.

Involvement in the interpretation of results will include a chance to provide feedback on results produced using the operational framework created by the Lay and Expert Consultation Groups, and to discuss the meaning and relevance of our findings for the wider British Pakistani community, as well as South Asian culture in a broader sense.

We will achieve this aim by meeting the following Objectives:

1. Establish a Lay Consultation Group consisting of two sub-groups of 10 male and female British Pakistani adults aged 18+ normally resident in Bradford, with
  - The first sub-group comprising 1^st^ and 2^nd^ generation British Pakistanis who frequent local community centres and mosques in Bradford, with separate meetings for men and women
  - The second sub-group comprising 3^rd^ and 4^th^ generation British Pakistanis who are students at the University of Bradford, with separate meetings for men and women
2. With each sub-group, hold focus group discussions during the Study Design stage to
  - Guide members to define, conceptualise and develop a categorisation / ranking of biraderi from a lay perspective
  - Obtain members’ reflections on previous meeting and collect feedback on the 1^st^ stage of the Delphi process and, if possible, contribute to a consensus schema of biraderi
3. During the Interpretation of Results stage, hold meetings with each sub-group to
  - Share overall results from the statistical analysis presented in simple sentences or easy-to-understand graphs
  - Obtain members’ feedback on how to interpret findings
  - Provide members an opportunity to suggest ways to improve child nutrition based on the study’s findings

### Participants

Membership of the Lay Consultation Group will be open to adults 18+ years old who are ordinarily resident in Bradford and identify as British Pakistani. Members must have some understanding of the biraderi system and know which biraderi group they belong to. The group will be sub-divided based on generation. The Lay Group will include a total of 40 members.

Each sub-group must comprise male and female members, preferably in equal numbers, with male-only and female-only meetings conducted to reflect cultural gender norms for older generations, and to mirror the process of gender-segregated discussions in younger generations. We would like to have participants from different generations of British Pakistanis, both male and female, who have been resident in Bradford for at least 10 years. We feel that 10 participants in each focus group session would be a manageable number.

It is expected that members of sub-group 1 will be older than members of sub-group 2, and that sub-group 1 will be drawn from community groups and mosques in Bradford, whereas sub-group 2 will be drawn from the student population of the University of Bradford (Table 1).

**Table 1:** Proposed composition of the Lay Consultation Group.

Generation	n	Definition	Simple term
**Sub-group 1**			
1^st^ Generation British Pakistani	10	Individuals who were born in Pakistan and settled in the UK in adulthood	Pakistan-born individuals
2^nd^ Generation British Pakistani	10	Individuals who were born in the UK to one or both parents who were born in Pakistan	Children of Pakistan-born individuals
**Sub-group 2**			
3^rd^ Generation British Pakistani	10	Individuals who were born in the UK to one or both parents also born in the UK and one or more grandparents born in Pakistan	Grandchildren of Pakistan-born individuals
4^th^ Generation British Pakistani	10	Individuals born in the UK with at least one parent and one grandparent also born in the UK but whose great-grandparents were born in Pakistan	Great-grandchildren of Pakistan-born individuals

### Recruitment

We will use two recruitment strategies, snowball and purposive sampling [34]. This is because participants may be difficult to reach (1) due to the sensitive nature of biraderi, and (2) because older 1^st^ generation immigrants will be difficult to locate. First, we will approach British Pakistani community groups and mosques in Bradford where the study team has working relationships with staff and students and ask them to circulate the project flyer and make an announcement about our study at a meeting. Second, we will recruit 3^rd^ and 4^th^ generation British Pakistanis from the University of Bradford students and circulate flyers in student union spaces and the refectory, as well as notice boards on campus. Once contact with a potential student participant has been made, we will use snowball sampling by asking them to share information about our study with any members of their family or friendship networks, especially of the 1^st^ or 2^nd^ generation.

If individuals agree to join one of the Lay Consultation sub-groups, the researchers will ask for confirmation that the participant is independently volunteering to take part in the focus groups and consent to being audio-recorded. They will also be sent a Participant Information Sheet in English and / or Urdu. The recruitment period will run from 1 June 2024 to 1 June 2025.

### Consent

Prior to the first focus group, participants will be asked to sign a Consent Form. We will translate the Participant Information Sheet, Consent Form, and Withdrawal Form into Urdu for those who do not speak English as their first language. Participants must be able to independently read and understand the consent documents to participate. Participants will be able to withdraw from the study at any time prior to, during, or after the focus group. However, they will also be notified that the research will not be able to extract their data from focus group recordings and transcripts. This information will be outlined on the consent form, and verbally stated at the start of the focus-group. The withdrawal criteria will allow participants to withdraw data up to two weeks after participating in the focus groups. They need to inform the researchers of their decision and complete a Participant Withdrawal Form. The researcher will also be able to automatically withdraw participants if there are any concerns or participants display unusual behaviour during the focus group. To keep track of such situations, a withdrawal form will be used to indicate when a participant has been withdrawn.

### Methods

We will collect data from the Lay Consultation Group in a series of focus groups. There will be three rounds of meetings for each sub-group, with a total of 12 meetings held over the course of the project.

Round 1 will be held during the design stage, and sub-groups will be tasked with creating a ranking of the 15 biraderi groups in the Born in Bradford study. In Round 2, also in the design stage, sub-groups will give feedback on the ranking proposed by the Expert Consultation Group and the analysis plan. After this, there will be a break of up to 6 months while the study investigators implement the categorisation and ranking schema in the planned analysis of Born in Bradford Data. In Round 3, during the post-analysis stage, sub-groups will give feedback on study results.

Focus groups will be audio-recorded, with additional note-taking on a laptop to support discussions and analysis. Recordings will be transferred to secure storage and deleted within 24 hours. Transcripts and notes will be combined for thematic data analysis, and ranking or ordering data will be presented in tables.

The topic guides and protocol for focus groups are attached separately.

## 4. Secondary data analysis—Born in Bradford

### Participants

The Born in Bradford study is an epidemiologic cohort of 12,453 women with 13,776 pregnancies booked at the Bradford Royal Infirmary maternity unit between 2007 and 2011. Nearly half of the cohort’s 6900 (50%) participants who are of South Asian origin have ties to Mirpur district in Azad Kashmir province in northern Pakistan. BiB includes a combination of routine data, Biobank, and research data collected using examination and questionnaires [14]. A BiB 1000 sub-cohort of 1763 children was followed up between 6 months and 4 years of age for repeated measurement of growth, diet, and other risk factors for childhood obesity [35]. Most recently, between 2017 and 2021, over 9000 children aged 7–11 years were followed up in the BiB Growing Up wave [36].

We will use data collected from the BiB Baseline, the BiB 1000 wave, BiB Growing Up, and some socioeconomic variables from routine information linked to individual records. Preliminary exploration of data availability (https://borninbradford.github.io/datadict/) shows that, of the 4042 biraderi records in the BiB Baseline Mother survey administered in pregnancy, it is possible to link to records on breastfeeding at 6 months (n = 760) and children’s total energy intake at 18 months (n = 690) from BiB 1000 data, and link biraderi to children’s self-reported food consumption at 7–11 years (n = 2142) from BiB Growing Up. If our consultation phase indicates the need for a ‘reference’ or comparator group comprising those who are not of South Asian origin or additional nutrition-related indicators (e.g., maternal food insecurity in pregnancy) essential for investigating biraderi-based inequalities, we will include a larger proportion of BiB participants and other relevant analysis variables.

We will access BiB datasets from 1 July 2024, and data will not contain information that could identify individual participants.

### Nutrition, diet, and socioeconomic position variables from BiB

Data from BiB 1000 have been analysed extensively, and we will replicate coding strategies, cut-offs and summary measures for breastfeeding [17], complementary feeding [16], and parental feeding styles [37, 38] when describing diet and nutrition-related outcomes. This will ensure that our findings can be compared to previous research from the BiB cohort and contextualised within wider public health nutrition frameworks.

Data from BiB Growing Up are still under embargo and open-ended responses on children’s self-reported daily food consumption have not yet been coded. We will work closely with the BiB team to code responses and derive nutritional values for each participant in Stage 1 and use this to describe children’s total calorie intake as well as their consumption of junk food or ultra-processed food. If we are not able to carry out this coding due to data quality issues, we will use another questionnaire item on how often children have a meal from a takeaway, employing this variable as a crude indicator of diet quality between 7 and 11 years.

We will use BiB socioeconomic position data on maternal and paternal factors (education and income) and neighbourhood conditions (Index of Material Deprivation and Lower Layer Super Output codes) as covariates or effect modifiers, using methods for coding and summarising used in previous BiB studies [39, 40]. Data on neighbourhood conditions will be used to construct a variable encoding geographic segregation of households by biraderi.

### Biraderi variables

The BiB Baseline Mother survey includes information on biraderi for both parents and all four grandparents. Since biraderi is largely patrilineal, we will use information on the index child’s paternal biraderi reported by the mother as the main analysis variable. The BiB Baseline Father survey also asks about biraderi, but this is a subset of only 3499 fathers of whom a proportion are of South Asian origin, compared to c. 4042 mothers who have provided information on biraderi for themselves and the child’s father in the Mother survey. We will cross-check a subset of proxy- and self-reported paternal biraderi data to ensure the data we use are accurate.

The BiB biraderi variable encodes 15 groups (*Jatt*, *Pathan*, *Rajput*, *Choudhry*, *Bains*, *Kashmiri*, *Mughal*, *Syed*, *Malik*, *Gujjar*, *Qureshi*, *Awaan*, *Sheikh*, *Qasabi*, and *Bains Rajput*), as well as ‘*Other’*, ‘*Don’t Know’*, and ‘*Missing*’ codes. Following a conceptual review, we will map biraderi groups reported in BiB onto existing biraderi schema in the literature (for example, Werbner’s hierarchy of *zat /* biraderi [6]) and use these to guide expert and lay consultations.

### Data linkage and missing data

We expect varying levels of missing information for biraderi, nutritional, and socioeconomic variables. Response rates will be captured fully during data access and analyses (Stages 1 and 3), and it is possible that we may not be able to link data for all sweeps from birth to 11 years. However, since each analysis will include data collected at baseline and at least one other time point, we will investigate missing data patterns, missing value patterns, and non-response mechanisms that affect longitudinal studies [41]. We will investigate these missing data mechanisms to assess whether using subsets of the cohort would lead to biased estimates in the event of covariate-induced differential non-response [42, 43]. For BiB 1000 data, we will attempt to use data from any or all available follow-up visits (at 6, 12, 18, 24 and 36 months), as this sub-cohort has intermittent non-response with c. 75% response rates at each sweep [14]. We will conduct complete case analyses and do not intend to use multiple imputation to deal with missing information.

### Statistical analysis

We will conduct and report analyses using the STROBE checklist [44].

We will first examine biraderi-specific infant and child nutrition outcomes in tables and graphs to identify underlying patterns and heterogeneity. Next, we will conduct explanatory analyses using variables for ordered social groups encoding our three sets of biraderi rankings.

It is possible that lay members will not see the need to rank biraderi groups, but we expect that the expert group will, since at least one scheme of hierarchy already exists in the literature [6]. We will extend our analyses of simply reporting group-differences to look at inequities, to understand whether so-called ‘lower’ ranking groups experience worse nutrition. A priori, we expect to investigate three pathways (Table 2).

**Table 2:** Proposed analyses of biraderi-related inequities in diet and nutrition outcomes.

Pathway	Description of pathway	Metrics / statistical technique
Graded inequality and the “sticky floor” [22, 45]	Diet and nutrition worsen with decreasing biraderi-group rank, with the largest gap for the so-called ‘lowest’ group	Absolute Concentration Index and Relative Concentration Index [21] to depict biraderi-related disparities in graphs and tables
Enclave effects [22, 46]	Individuals in a biraderi group have better diets and nutrition when they live in neighbourhoods where they form the largest biraderi group (own enclave) than when they are a minority.	Regression models with biraderi as a predictor, stratified by ‘own enclave’.
Network effects [22, 45]	Biraderi is related to nutrition within the overall pattern of socioeconomic disparities, such that higher income households of a so-called higher-ranking biraderi have better outcomes.	Regression models with terms for statistical interaction between socioeconomic position and biraderi

## Ethical considerations

Since this project is mainly secondary data analysis of the Born in Bradford cohort, many ethical issues are already addressed by the original study regarding informed consent, confidentiality and anonymity, and prompt referral to appropriate services for participants at high-risk of ill health during data collection, with additional safeguards put in place by the Born in Bradford team for data access, storage, transfer and use. We will abide by data protection requirements outlined by the Born in Bradford study. In addition, their governance and oversight processes involve formal review of draft manuscripts of results, ensuring that they can provide input on wording of results or terminology to ensure our published output responsibly reports a topic that may be sensitive to their study population.

Therefore, our main ethical concerns relate to user involvement in the Expert and Lay Consultation Groups, and ethical interpretation and reporting of study findings following secondary data analysis of the Born in Bradford cohort.

### Sensitive nature of the research topic

The contentious, politicised, and polarising debate around caste / biraderi can contribute to stigma and discrimination for an individual who reveals this aspect of their identity or is ’unmasked’ by another. This is a particular risk for our Lay Consultation Group members recruited from the British Pakistani population of Bradford, whose caste / biraderi could be revealed simply by stating their surnames. We will mitigate this risk in two ways: (i) drafting Terms of Reference for the Lay Consultation Group emphasising that members keep details of group composition confidential and do not post any information or images of the group on social media; and (ii) not listing the names of the Lay Consultation Group in any study outputs or publications. The composition of the Expert Consultation Group will remain anonymous, as is common in Delphi studies.

The Lay and Expert groups will be given information sheets and consent forms prior to participation, and requested to adhere to a Terms of Reference document upon enrolment (Lay Group only). Qualitative data from online (Expert Group) and in-person (Lay Group) consultations will be anonymised for analysis, but given the sensitive nature of the topic and possible risks to anonymity, we will not share data publicly. Quantitative data comprising ranking tables will be collected at the group level without information attributable to individual members, and this will be published in summary form.

The two co-investigators, Dr Akhtar (Aston University, Birmingham) and Dr Intezar (University of Bradford) have extensive experience in conducting primary research, interviews and group discussions with Muslim communities in Bradford, and they will lead or guide the process to ensure participants have opportunities to ask questions and maintain anonymity, confidentiality and respectful interaction.

A further ethical issue relates to the risks associated with findings that point to inequalities within British Pakistani groups that may further stigmatise and marginalise them due to the perceived cultural practices or beliefs that shape hierarchies. We will mitigate this by involving Lay and Expert groups in framing the key messages of our project to ensure we reduce the risk of backlash and have a constructive dialogue through dissemination. Dr Akhtar is also particularly experienced in discussing issues around politics and identities among British Pakistanis and will provide guidance on the best way to adopt sensitive communication strategies for our epidemiological findings.

### Compensation for participants

Participants in the Lay Consultation Group will be offered a gift voucher for participating in focus groups.

Participants in the Expert Consultation Group will be offered the chance to participate in a free academic workshop hosted towards the end of the project. They can choose to sign up for a notification about the email even if they decline participation in the Expert Consultation Group at the recruitment stage. Participants in the Expert Consultation Group will not be offered a monetary incentive due to the difficulty of making many payments internationally for small amounts in multiple currencies. For equality, UK-based experts will not receive a payment or gift either.

## Dissemination plan

We anticipate several academic and non-academic outputs with related impacts from this project (Table 3), as well as further engagement opportunities through invited talks and seminars during the project.

**Table 3:** Proposed academic, policy and community outputs.

Output	Expected impacts
(1) **Journal article**: Interdisciplinary review of biraderi literature	Contribute to conceptual literature on health inequalities
(2) **Journal article:** Reporting Delphi and Lay Consultation exercises	(1) Improve reproducibility of findings (2) Methodological development in social epidemiology
(3) **Journal article**: Results of statistical analyses	(1) Findings of scientific importance for public health (2) Policy-relevant findings on child health in the UK
(4) **Conference paper**: Society for Social Medicine and Population Health Annual Scientific Meeting	Contribute to discussions on social epidemiology and causal inference in public health
(5) **Conference paper:** International Conference on Nutrition and Growth	Contribute to emerging evidence and debates on nutritional inequalities in infancy and childhood
(6) **Academic workshop:** Hosted online for social epidemiology and caste / biraderi researchers	Increase visibility of our innovative research methods for a broader international community of researchers interested in biraderi / caste
(7) **Policy output**: podcast hosted by UCL or Aston University	Summarise implications of findings for lay audiences
(8) **Policy output**: article in *The Conversation*	Summarise study methods and findings for non-specialist readers
(9) **Community**: panel discussion at Bradford 2025 event or Bradford Literature Festival	Highlighting the importance of equality in child nutrition outcomes within the wider urban development agenda for Bradford post-2025
(10) **Community**: research exhibition in local mosque, community centre or university	Provide Bradford residents with insight into patterns of child nutrition in the local community and highlight areas for action and intervention

## Discussion

Our study addresses research questions related to child health and nutrition, directly in the UK, but also indirectly in any part of the world where children experience disadvantage linked to sociocultural hierarchy and identity. Our findings will be of particular use in (i) identifying women and children at particular risk of suboptimal breastfeeding practices, poor complementary feeding, and unhealthy diets in primary school in the UK, and (ii) elucidating the sociocultural pathways through which inequalities in population health nutrition outcomes are expressed. Given that not breastfeeding costs 0.5% of world gross national income [47] and the wider costs of obesity to UK society exceed £27 billion annually [48], our research could help solve real world problems with vast social and economic impacts.

The BiB cohort is unique in that its core observational research is complemented with applied health research to prevent childhood obesity [49]. Our findings will be relevant for the BiB team’s intervention research. Additionally, ours will be the first study to code data on children’s self-reported food consumption at 7–11 years of age and derive nutritional intakes, creating a valuable resource for researchers interested in child nutrition in primary school. The BiB team’s support for our study will enable us to link with their ongoing programme of work.

Internationally, there is a large network of researchers interested in caste / identity-based inequalities in social and economic outcomes in South Asia, as well as social epidemiologists interested in the causal effects of other types of identity such as race and ethnicity. We will create links through conference attendance and use our Expert Consultation Group to ‘snowball’ our efforts.

Our study has the potential to influence all public health programming in the UK that aims to address ethnic inequalities. Findings will also provide valuable insight for local community-based groups that address health, wellbeing, culture, and diversity in Bradford (e.g., Better Start Bradford, Bradford & District Community Empowerment Network, Impact Hub Bradford, and Amal).

In addition to being the first study to look at biraderi-based nutritional inequalities in the UK, our study is innovative in that we will formally involve experts and users in the design and interpretation of our quantitative analyses. Participatory approaches are common in intervention and implementation research [50], but our study will be one of the first to do this in the context of planning and conducting secondary data analyses, echoing recent methodological developments in biosocial ethnography applied to cohort studies [51].

Our research is well-timed in that we will disseminate our findings in the first six months of 2025, a year of activities for Bradford as the UK City of Culture 2025 (https://bradford2025.co.uk/). This presents an opportunity for us to hold a community event to discuss inequalities in children’s diets in Bradford. Further, by presenting findings from the most recent wave of BiB Growing Up data on primary school children, our research will contribute to ongoing health advocacy, policy and programme efforts in the city to address childhood obesity.
