# Correction: Cultural hierarchies in health: Does inherited sociocultural position (biraderi) shape diet and nutrition among British Pakistani children? Protocol for a mixed-methods study

**DOI:** 10.1371/journal.pone.0313790

**Published:** 2024-11-08

**Authors:** The PLOS ONE Editors

## Notice of Republication

This article was republished on November 1, 2024, to address an issue identified post-publication. The publisher apologizes for the error. Please download this article again to view the correct version.
