# Supplementary material for: Cultural hierarchies in health: Does inherited sociocultural position (biraderi) shape diet and nutrition among British Pakistani children? Protocol for a mixed-methods study
Source: PLoS One. 2024 Jun 17;19(6):e0305556. doi: 10.1371/journal.pone.0305556 (PMC11182522; doi:10.1371/journal.pone.0305556)
Supplement: Peer review history

# Editor report: Decision Letter 0

**DOI:** 10.1371/journal.pone.0305556.r001

**Authors:** Avanti Dey

29 May 2024

Cultural hierarchies in health: Does inherited sociocultural position (biraderi) shape diet and nutrition among British Pakistani children? Protocol for a mixed-methods study.

PONE-D-24-20329

Dear Dr. Bhatia,

We’re pleased to inform you that your manuscript has been judged scientifically suitable for publication and will be formally accepted for publication once it meets all outstanding technical requirements.

Within one week, you’ll receive an e-mail detailing the required amendments. When these have been addressed, you’ll receive a formal acceptance letter and your manuscript will be scheduled for publication.

An invoice will be generated when your article is formally accepted. Please note, if your institution has a publishing partnership with PLOS and your article meets the relevant criteria, all or part of your publication costs will be covered. Please make sure your user information is up-to-date by logging into Editorial Manager at Editorial Manager® and clicking the ‘Update My Information' link at the top of the page. If you have any questions relating to publication charges, please contact our Author Billing department directly at authorbilling@plos.org.

If your institution or institutions have a press office, please notify them about your upcoming paper to help maximize its impact. If they’ll be preparing press materials, please inform our press team as soon as possible -- no later than 48 hours after receiving the formal acceptance. Your manuscript will remain under strict press embargo until 2 pm Eastern Time on the date of publication. For more information, please contact onepress@plos.org.

Kind regards,

Avanti Dey, PhD

Staff Editor

PLOS ONE

Additional Editor Comments (optional):

We have deemed your submission eligible for expedited acceptance based on the documents provided, and the quality of your work. Thank you for submitting your work to PLOS ONE!

Reviewers' comments:
